# Post-Processing Bias Field Inhomogeneity Correction for Assessing Background Parenchymal Enhancement on Breast MRI as a Quantitative Marker of Treatment Response

**DOI:** 10.3390/tomography8020072

**Published:** 2022-03-22

**Authors:** Alex Anh-Tu Nguyen, Natsuko Onishi, Julia Carmona-Bozo, Wen Li, John Kornak, David C. Newitt, Nola M. Hylton

**Affiliations:** 1 Department of Radiology and Biomedical Imaging, University of California San Francisco, San Francisco, CA 94143, USA; aanguyen@seas.upenn.edu (A.A.-T.N.); julia.carmonabozo@ucsf.edu (J.C.-B.); wen.li@ucsf.edu (W.L.); dnewitt@sbcglobal.net (D.C.N.); nola.hylton@ucsf.edu (N.M.H.); 2 Department of Epidemiology and Biostatistics, University of California San Francisco, San Francisco, CA 94143, USA; john.kornak@ucsf.edu

**Keywords:** bias correction, breast cancer, breast MRI, background parenchymal enhancement, neoadjuvant chemotherapy

## Abstract

Background parenchymal enhancement (BPE) of breast fibroglandular tissue (FGT) in dynamic contrast-enhanced breast magnetic resonance imaging (MRI) has shown an association with response to neoadjuvant chemotherapy (NAC) in patients with breast cancer. Fully automated segmentation of FGT for BPE calculation is a challenge when image artifacts are present. Low spatial frequency intensity nonuniformity due to coil sensitivity variations is known as bias or inhomogeneity and can affect FGT segmentation and subsequent BPE measurement. In this study, we utilized the N4ITK algorithm for bias correction over a restricted bilateral breast volume and compared the contralateral FGT segmentations based on uncorrected and bias-corrected images in three MRI examinations at pre-treatment, early treatment and inter-regimen timepoints during NAC. A retrospective analysis of 2 cohorts was performed: one with 735 patients enrolled in the multi-center I-SPY 2 TRIAL and the sub-cohort of 340 patients meeting a high-quality benchmark for segmentation. Bias correction substantially increased the FGT segmentation quality for 6.3–8.0% of examinations, while it substantially decreased the quality for no examination. Our results showed improvement in segmentation quality and a small but statistically significant increase in the resulting BPE measurement after bias correction at all timepoints in both cohorts. Continuing studies are examining the effects on pCR prediction.

## 1. Introduction

Dynamic contrast-enhanced magnetic resonance imaging (DCE-MRI) provides quantitative measurements reflecting the contrast enhancement kinetics of lesions and normal tissues. Breast DCE-MRI measurements may be helpful to understand tumor biology and physiology in breast cancer patients. More specifically, signal enhancement observed in breast fibroglandular tissue (FGT) following contrast injection is known as background parenchymal enhancement (BPE). BPE has been shown to be associated with breast cancer risk and can be used as an imaging biomarker [1]. In clinical practice, BPE is qualitatively interpreted by a radiologist according to the Breast Imaging Reporting and Data System (BI-RADS) atlas using four categories: minimal, mild, moderate, or marked [2]. Studies based on qualitatively assessed BPE are subject to inter-reader variability, which limits its use as an imaging biomarker. As a result, numerous studies have investigated quantitative approaches for measuring BPE and found it to be correlated with breast cancer risk, treatment response, and outcome [3,4,5,6,7,8,9,10,11,12,13,14]. Currently, there is no standardized method for calculating quantitative BPE and many studies differ in their algorithms. For example, van der Velden et al. calculates the late phase mean BPE in the top 10% of voxels exhibiting enhancement [7,9] whereas Wu et al. calculates the absolute and relative volume of enhanced FGT above a predefined enhancement threshold at early and delayed phases [11]. We recently reported a fully automated method of calculating contralateral BPE by taking the mean percent enhancement of FGT voxels at the early phase [12].

The quality of FGT segmentation can affect BPE quantification. However, accurate segmentation of breast FGT using fully automated methods is a challenge when image artifacts are present. Low spatial frequency intensity nonuniformity due to coil sensitivity variations seen in the MRI data is known as bias or inhomogeneity, directly relating to image quality. Thus, the presence of bias field inhomogeneity can negatively impact the quantification of BPE which may be particularly problematic in multi-center trials utilizing multiple imaging platforms. To address this obstacle, we utilized the N4ITK algorithm, an improvement over the N3 (nonparametric nonuniformity normalization) method [15], to perform bias correction. N4 bias correction has improved B-spline fitting and a modified iterative optimization scheme which improves convergence performance. It is an intensity distribution-based method that starts by iterating through deconvolving the intensity histogram by a Gaussian, remapping the intensities, and then spatially smoothing the result using a B-spline model until we reach our convergence threshold or a maximum number of iterations.

The I-SPY 2 TRIAL (Investigation of Serial Studies to Predict Your Therapeutic Response through Imaging and Molecular Analysis 2, NCT01042379) is an ongoing multicenter clinical trial. This trial included a total of 25 participating sites with different magnetic resonance scanner vendors, models, configurations, and sequences. MRI examinations had various levels and types of field inhomogeneity across the participating sites. For this retrospective study, we tested the effect of applying the N4ITK algorithm over a restricted volume encompassing both breasts prior to BPE measurement. We applied our automated breast FGT segmentation method to uncorrected and bias-corrected MRI and performed a comparative visual assessment of the two FGT segmentations. We also quantitatively compared the two FGT segmentations and the resulting BPE measurements.

## 2. Materials and Methods

### 2.1. Study Cohort

**Figure 1:**
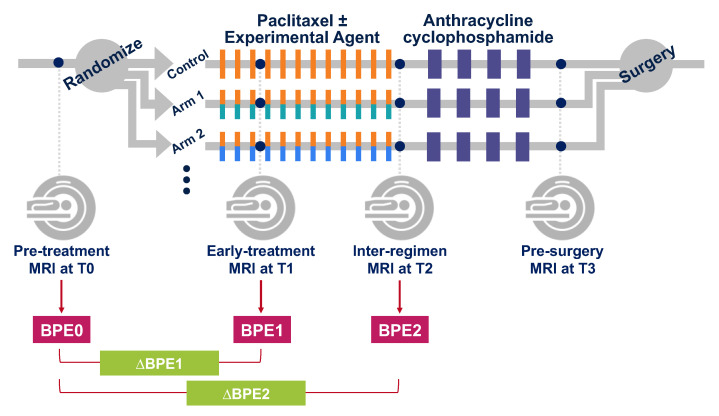
I-SPY 2 study schema with adaptive randomization.

This retrospective study is based on the MRI data of 990 breast cancer patients who were enrolled and randomized to neoadjuvant chemotherapy (NAC) drug arms in the I-SPY2 TRIAL from May 2010 to November 2016. Women older than 18 years of age diagnosed with locally advanced breast cancer (tumor size ≥ 2.5 cm) were eligible to enroll in this multicenter clinical trial. Patients with evidence of distant metastasis and patients with tumors that were diagnosed as HR+/HER2− and low risk according to the MammaPrint 70-gene assay were excluded from the trial. Figure 1 shows the I-SPY2 trial schema. Patients were randomized to the control (paclitaxel for HER2− or paclitaxel and trastuzumab for HER2+) or one of the experimental drug arms. Participants received a weekly dose of paclitaxel alone (control) or in combination with an experimental agent for 12 weekly cycles followed by four cycles of anthracycline–cyclophosphamide (AC) before surgery. MRI exams were performed before the initiation of NAC (pre-treatment, T0), after 3 weeks of treatment (early-treatment, T1), after 12 weeks and between drug regimens (inter-regimen, T2), and after completion of NAC and prior to surgery (pre-surgery, T3). All patients provided written informed consent at the screening in order to participate in the trial. A second consent was obtained if the patient was randomized to an experimental treatment.

Of the 990 patients enrolled on completed drug arms of I-SPY 2 before November 2016, this study included 878 patients whose detailed surgical pathology including residual cancer burden was available as of December 2019. Of the 878 patients, we excluded 97 patients who did not have all four longitudinal DCE-MRI exams because of the following reasons: patient’s withdrawal of treatment consent, patient illness, missed patient appointments, MRI technical issues, or other image quality or protocol adherence issues. We also excluded 46 patients who had failed determination of breast contour using our automated method for at least one of the four longitudinal DCE-MRI exams.

After these preliminary exclusions, BPE was calculated in 735 women (median age, 49 years; range, 24–77) and were defined as the “whole cohort”, in which 258 (35.1%) patients achieved pCR. Of the 735 patients, 340 women (median age, 49 years; range, 24–77) were defined as the “high-quality cohort” based on the FGT segmentation quality as described in our previous publication [12] and later in this article (Section 2.7), in which 113 (33.2%) patients achieved pCR.

### 2.2. Pathological Response Assessment

Pathologic complete response (pCR) was defined as the absence of residual invasive cancer in the breast or lymph nodes at the time of surgery. All patients were classified as either pCR or non-pCR. Patients who did not complete the assigned treatment or did not undergo surgery for any reason were considered non-pCR.

### 2.3. MRI Data Acquisition

MRI data were acquired with 1.5T or 3T scanners using a dedicated breast RF coil, across a variety of vendor platforms and institutions. All MRI exams for the same patient were performed using the same magnet configuration (manufacturer, field strength, and breast coil model). The standardized image acquisition protocol included T2-weighted and DCE-MRI sequences performed bilaterally in the axial orientation. DCE-MRI was acquired as a series of 3D fat-suppressed T1-weighted images with the following parameters as specified in the I-SPY2 MRI protocol: TR = 4–10 ms, minimum TE, flip angle = 10–20°, field of view (FOV) = 260–360 mm to achieve full bilateral coverage, acquisition matrix = 384–512 with in-plane resolution ≤1.4 mm, and slice thickness ≤2.5 mm, temporal resolution = 80–100 s. Gadolinium contrast agent was administered intravenously at a dose of 0.1 mmol/kg body weight, and at a rate of 2 mL/s, followed by a 20 mL saline flush. The same contrast agent brand was used for all MRI exams for the same patient. Pre-contrast and multiple post-contrast images were acquired using identical sequence parameters. Post-contrast imaging continued for at least 8 min following contrast agent injection.

### 2.4. FGT Segmentation and BPE Calculation

Automatic whole breast segmentation was performed (whole breast mask) using in-house software developed in IDL (L3Harris Geospatial, Broomfield, CO, USA). Both breasts were initially segmented from background for the volumes anterior to the sternal notch using pre-contrast images reformatted to the coronal orientation. FGT of only the contralateral breast was then segmented using fuzzy c-means (FCM) clustering [16]. All clusters within the central 50% of all axial slices containing FGT were combined as a half-stack BPE mask as described in a previous study [12]. For our semi-automated FCM method, we explicitly set the number of clusters to 6 and we chose to keep the first six clusters as our tissue segmentation to differentiate between fat and tissue. The advantage to the applied fuzziness is that this addresses the partial volume effects happening when multiple tissues contribute to a single voxel. We set the number of iterations to 20 in the FCM algorithm in order to find the best solution. BPE was calculated by taking the mean percent enhancement (PE = (S_1 − S_0)/S_0 × 100%, where S_0 and S_1 are voxel-wise signal intensities at pre-contrast and early post-contrast phase, respectively) of all voxels in the half-stack BPE mask.

### 2.5. Bias Correction

**Figure 2:**
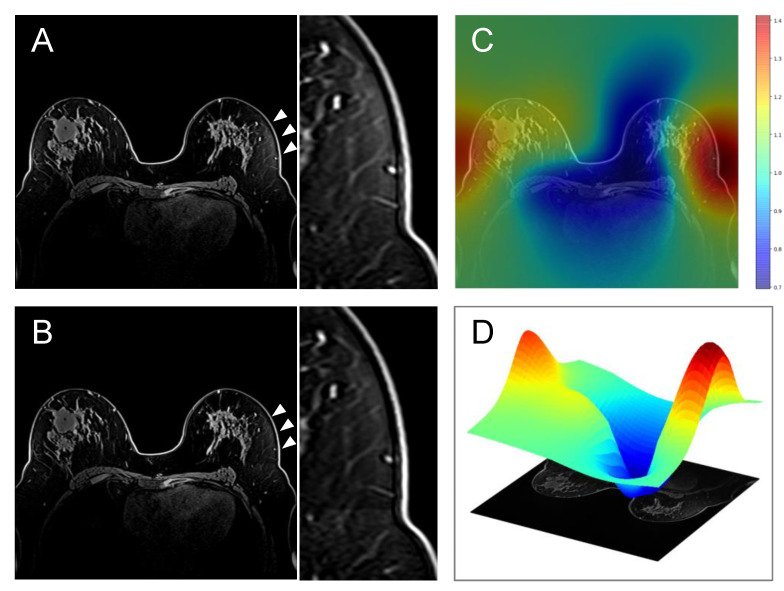
(**A**) Uncorrected pre-contrast image and the enlarged image, (**B**) Bias-corrected pre-contrast image and the enlarged image, (**C**) Overlay of the corresponding estimated bias field, (**D**) 3D surface plot of the estimated bias field on a single axial slice. The numbers (unitless) in the scale represent the distribution of pixel intensity mean and variance with respect to the measured tissue in the local region. Please note that (**C**,**D**) show the estimated bias field for the whole image. In this study, bias-correction was performed only within the whole breast mask. Arrowheads and the enlarged images highlight the area with significant field inhomogeneity in the contralateral breast.

To correct for image inhomogeneity, pre-contrast images for all exams were preprocessed with N4 bias correction within the generated whole breast mask. The N4 bias correction code was sourced from the Advanced Normalization Tools (ANTs) package developed by Avants et al. [17]. Default parameters were used and have been shown to work fairly well in a variety of applications, such as brain and lung [18,19]. Figure 2 shows an example of the estimated bias field and bias correction. A 3D surface plot of the estimated bias field is also shown for clarity and to show where the image is affected most by bias field inhomogeneity.

### 2.6. Quantitative Comparison of Uncorrected vs. Bias-Corrected BPE Masks

For each exam, two different BPE masks were generated from the uncorrected image (uncorrected BPE mask) and the bias-corrected image (bias-corrected BPE mask). For each BPE mask, the voxel count and the resulting BPE measurement were calculated. We also calculated the Sørensen-Dice similarity coefficient (DICE score) between the two BPE masks for each exam. The DICE score gives a quantitative overview of how much the FGT segmentation has changed after bias correction.

### 2.7. Visual Comparison of Uncorrected vs. Bias-Corrected BPE Masks

In our previous studies [12,14], radiologist 1 (N.O., a breast radiologist with 10 years of experience in breast MRI) visually assessed the quality of bias-corrected BPE mask using a 3-point grade (good, adequate, or poor) based on the presence and degree of under or oversampling because of coil artifacts, poor fat suppression or tissue distortion. The assessment was performed using a PDF report for each patient and each timepoint showing pre-contrast T1-weighted images with and without BPE mask overlaid at representative three slices in the axial orientation: the center slice and slices at the upper and lower ends of the half-stack BPE mask (BPE report). Based on the assessments, a high-quality cohort (*n* = 340) was identified from the whole cohort (*n* = 735) by limiting to patients with good or adequate FGT segmentations at all three time points of T0, T1 and T2 [12].

For the current study, a comparative visual assessment of uncorrected and bias-corrected BPE masks was performed by the same reader (radiologist 1, N.O.) after 2 years of the interval from the initial segmentation quality assessment. BPE reports were separately prepared for uncorrected and bias-corrected masks at same representative slices, labeled as A and B, respectively, and presented in a blinded randomized order. The radiologist reader assessed which BPE mask (A or B) showed better agreement with the visually observed distribution of the fibroglandular tissue on the corresponding slices of the pre-contrast T1-weighted image (gold standard) and whether there was a substantial difference between A and B equivalent to one or two grades (i.e., poor vs. adequate, adequate vs. good, poor vs. good). Concretely, the comparison was assessed using five categories as follows (A >> B: better agreement for A than B with change in grade, A > B: better agreement for A than B within grade, A = B: A and B show equivalent agreement, A < B: worse agreement for A than B within grade, A << B: worse agreement for A than B with change in grade). By collating the labels (A or B) for uncorrected and bias-corrected BPE masks which were blinded for the reader at the time of assessment, the above assessments were translated into five categories (−2, −1, 0, 1, and 2) as shown in Table 1. Negative values represent a worse agreement for the bias-corrected BPE mask than the uncorrected BPE mask, a zero represents that the two masks showed an equivalent agreement with the gold standard, and positive values represent a better agreement for the bias-corrected BPE mask than the uncorrected BPE mask. A 2 or −2 represents that the bias-corrected BPE mask showed better or worse agreement than the uncorrected BPE mask with a substantial difference.

To assess the inter-reader agreement of the comparative visual assessment, radiologist 2 (J.C.-B. a breast radiologist with 5 years of experience in breast MRI) independently assessed a sub-sample of 100 patients using the same method as radiologist 1.

### 2.8. Statistical Analysis

For the comparison of patient characteristics (the high-quality cohort vs. the non-high-quality patients), the Mann–Whitney U test for continuous variables and the Fisher exact test for categorical variables were used. Cohen’s weighted kappa between the two readers was estimated to evaluate the inter-reader agreement of the comparative visual assessment in the sub-sample of 100 patients. The voxel count and BPE measurements before and after bias correction were compared using Wilcoxon signed-rank test. To examine the performance of uncorrected and bias-corrected BPE measurements in predicting pCR, single predictor logistic regression models for pCR were developed independently for percent change in BPE from T0 to T1 (ΔBPE1) and from T0 to T2 (ΔBPE2) using Scikit-learn [20]. Hyperparameter optimization was performed using a grid search over the inverse of regularization strength [100, 10, 1.0, 0.1, 0.01] and optimization solver [‘newton-cg’, ‘lbfgs’, ‘liblinear’]. Bootstrap sampling with 2000 iterations was performed to create multiple training datasets with the out-of-sample data used each time as the corresponding test set. The area under the receiving operator curve (AUC) of the logistic regression hyperparameter optimized model was used to assess the predictive performance of ΔBPE1 and ΔBPE2 in the full patients and within sub-groups by immunohistochemical subtypes (HR+/HER2−, HR+/HER2+, HR−/HER2+, HR−/HER2−), independently within the whole cohort and the high-quality cohort.

## 3. Results

### 3.1. Patient Characteristics

Of the 990 patients enrolled at 22 clinical centers, 735 met inclusion for this study. Patient characteristics are shown in Table 2. No statistically significant differences were found in patient characteristics between the high-quality cohort (*n* = 340) and the non-high-quality patients (*n* = 395). Furthermore, the data summaries of all characteristics appear to be well-matched qualitatively between the cohorts.

### 3.2. Quantitatively Evaluated Effect of Bias Correction

Table 3 and Table 4 show the voxel count and BPE measurements for the two BPE masks before and after bias-correction in the whole cohort and the high-quality cohort, respectively. At all timepoints in both cohorts except for at T2 in the whole cohort, the estimated pseudo-median differences in the voxel counts between the two masks suggest potentially important change after bias-correction (approximately from 300 to 700 in absolute value). However, these were count changes corresponded to an average change that was not statistically significantly different from zero, and the corresponding confidence intervals included both negative and positive values above >100 in absolute value. The results from these data were, therefore, inconclusive regarding the question of whether voxel count increased or decreased on average after bias correction, with potentially clinically important effects being plausible in either direction (Table 3). After bias correction, small (but statistically significant) increases in BPE measurements were observed for all timepoints in both cohorts (Table 4). In the whole cohort, the median [first, third quartile] of DICE score were 0.846 [0.771, 0.895] at T0, 0.844 [0.761, 0.899] at T1, and 0.837 [0.763, 0.893] at T2. In the high-quality cohort, the median [first, third quartile] of DICE score were 0.866 [0.808, 0.907] at T0, 0.866 [0.807, 0.913] at T1, and 0.864 [0.800, 0.908] at T2. For all timepoints, higher median DICE scores were observed when restricted to only the high-quality cohort as opposed to when considering the whole cohort.

**Figure 3:**
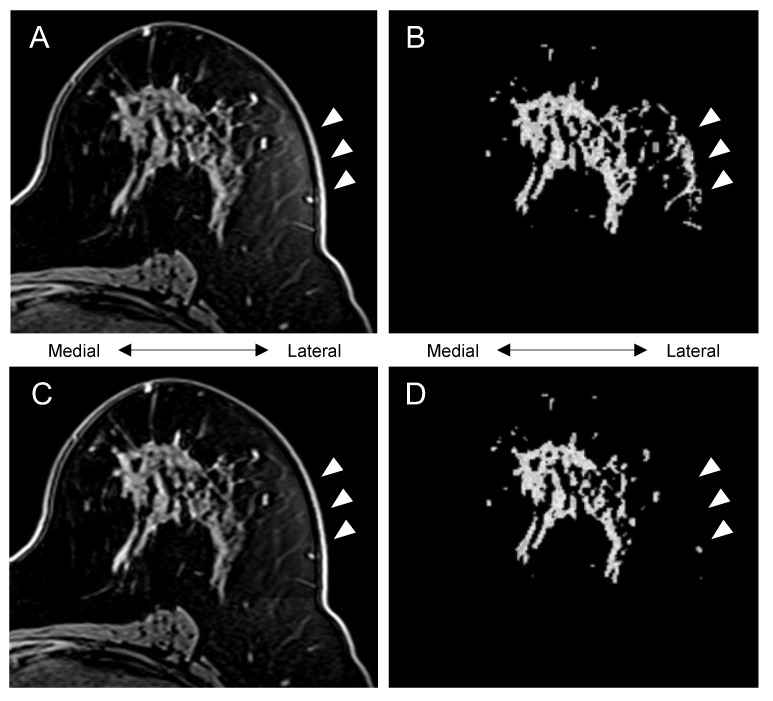
Representative section of a case: (**A**) uncorrected pre-contrast image, (**B**) uncorrected BPE mask (voxel count, 79492; BPE measurement, 42.7), (**C**) bias-corrected pre-contrast image, (**D**) bias-corrected BPE mask (voxel count, 61205; BPE measurement, 45.0). Arrowheads highlight the effect of bias-correction.

In Figure 3, an example of bias field inhomogeneity is shown. In the uncorrected pre-contrast image (top left), evident bias field inhomogeneity is seen on the lateral area of the breast and the uncorrected BPE mask (top right) includes that area. In the bias-corrected pre-contrast image (bottom left) and the bias-corrected BPE mask (bottom right), bias field inhomogeneity and its inclusion within the segmentation are alleviated.

**Figure 4:**
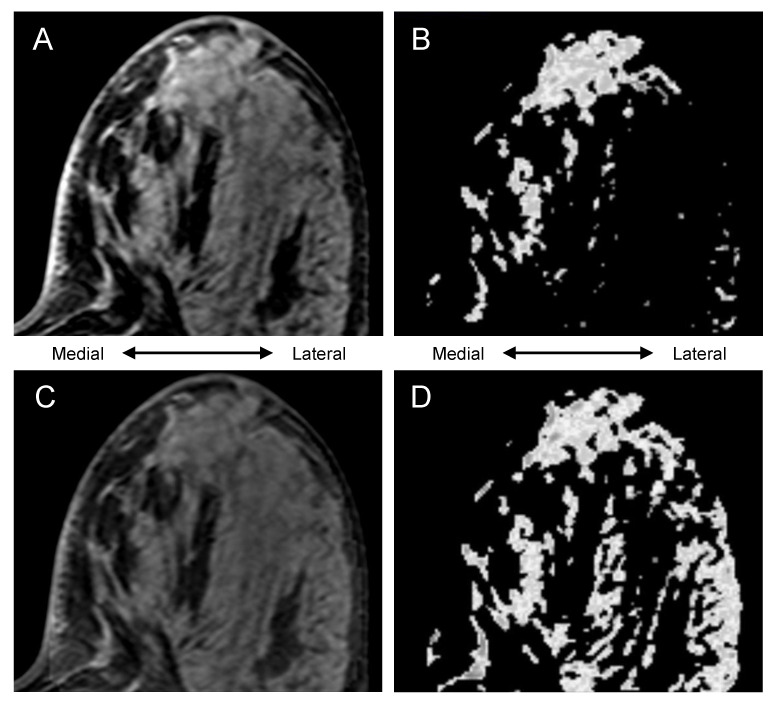
Representative section of a case: (**A**) uncorrected pre-contrast image, (**B**) uncorrected BPE mask (voxel count, 145049; BPE measurement, 24.0), (**C**) bias-corrected pre-contrast image, (**D**) bias-corrected BPE mask (voxel count, 186266; BPE measurement, 32.5).

In Figure 4, the uncorrected BPE mask (top right) failed to include the lateral area of the fibroglandular tissue. Because of the bias field inhomogeneity as shown in the uncorrected pre-contrast image (top left), it is assumed that the automated FCM clustering classified the medial and the lateral part of the fibroglandular tissue as different clusters, which led to the apparent under-segmentation. In the bias-corrected pre-contrast image (bottom left), bias field inhomogeneity is alleviated, and the bias-corrected BPE mask (bottom right) successfully included the lateral area.

### 3.3. Visually Evaluated Effect of Bias Correction

**Figure 5:**
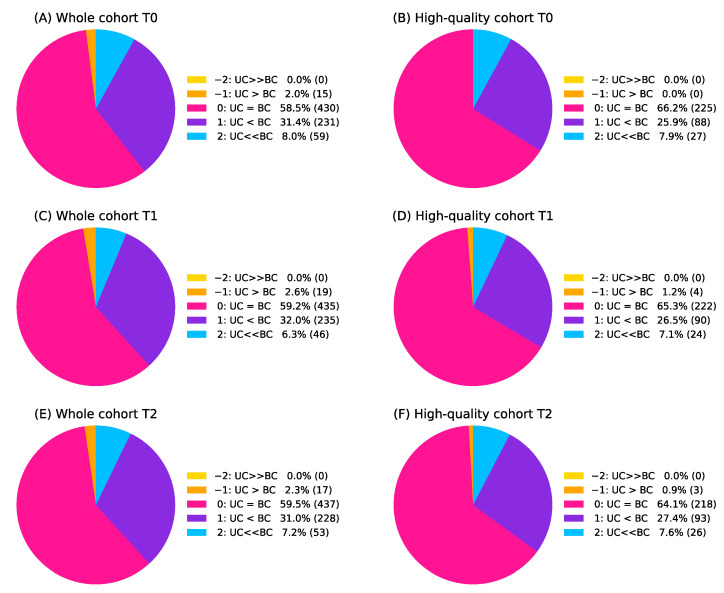
(**A**–**F**) Results for comparative visual assessment of uncorrected (UC) and bias-corrected (BC) BPE masks at each timepoint in the whole cohort and the high-quality cohort. Detailed explanations for the five categories (−2, −1, 0, 1, 2) can be found in Table 1. Numbers in the parentheses are the number of examinations.

Figure 5 shows the results for the comparative visual assessment of uncorrected and bias-corrected BPE masks at T0, T1, and T2 in the whole cohort and the high-quality cohorts. At all timepoints in both cohorts, 6.3–8.0% of examinations showed substantially better agreement with the gold standard for the bias-corrected BPE mask than the uncorrected BPE mask (category 2), while no examinations showed substantially worse agreement with the gold standard for the bias-corrected BPE mask than the uncorrected BPE mask (category −2). Only 0–2.6% of examinations were categorized as −1 (worse agreement for the bias-corrected BPE mask than the uncorrected BPE mask). The two categories with the largest sets of the examinations were categorized as 0 (58.5–66.2%, equivalent agreement with the gold standard) and 1 (25.9–32.0%, better agreement for the bias-corrected BPE mask than the uncorrected BPE mask). In the sub-sample assessment by radiologist 2, similar results were shown. Between radiologist 1 and 2, the Cohen’s weighted kappa coefficient for the comparative visual assessment was 0.58 [95% CI: 0.41, 0.76] for examinations at T0, 0.53 [95% CI: 0.32, 0.73] for examinations at T1, and 0.53 [95% CI: 0.34, 0.72] for examinations at T2.

3.4. pCR Prediction Analysis

**Figure 6:**
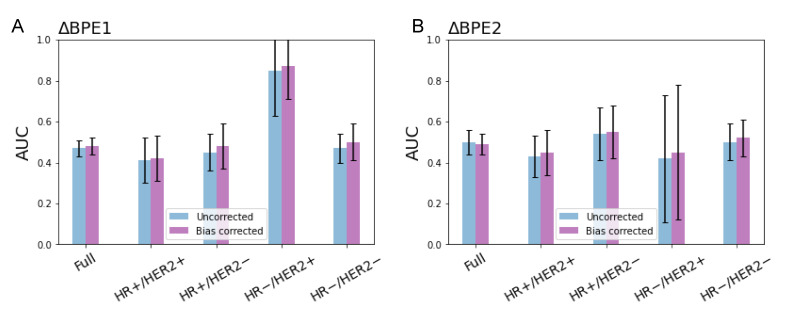
pCR prediction performance of BPE in the high-quality cohort (*n* = 340): (**A**) percent change in BPE from T0 to T1 (ΔBPE1) and (**B**) percent change from T0 to T2 (ΔBPE2). Error bars show 1 standard deviation.

For both the whole cohort and the high-quality cohort, logistic regression models using percent change of uncorrected and bias-corrected BPE as predictor showed similar predictive results with a large overlap in the full cohort and within sub-groups by immunohistochemical subtypes. Still, it is noteworthy that all sub-groups in the high-quality cohort showed higher estimated mean AUC for the bias-corrected BPE than the uncorrected BPE both in ΔBPE1 and ΔBPE2 in this study cohort (Figure 6).

## 4. Discussion

This study examined whether bias correction performed over a restricted bilateral volume on breast DCE improved segmentation of fibroglandular tissue and resulting measurement of BPE for prediction of a pathologic response to neoadjuvant chemotherapy. Bias-correction substantially increased the fibroglandular tissue segmentation quality for 6.3–8.0% of examinations, while it substantially decreased the quality for no examination. Our results showed a small but statistically significant increase in BPE measurements after bias correction at all timepoint in both cohorts. Percent change of BPE based on uncorrected and bias-corrected BPE masks showed similar predictive performance of pCR.

Biomarkers play an important role in the management of patients with breast cancer. In particular, biomarkers based on gene signatures are increasingly being used to predict treatment response and patient outcome. This helps to estimate the optimal treatment strategy and provide precision medicine. Previous studies have shown that BPE derived from breast MRI has promise as an imaging biomarker to predict treatment outcome in NAC for breast cancer [3,4,12,13,14,21,22,23,24,25]. In order to obtain the most accurate BPE measurements and the prediction based on them, a high standard of image processing must be met. At the same time, an automated method of quantitative calculation is essential in order to realize clinically applicable BPE measurement workflow [1,25,26]. Thus, we have been investigating a robust, automated method to quantitatively assess BPE [16,27]. One of the biggest challenges that we experience in the multi-center setting is the presence of image artifacts that can negatively impact the quality of automated FGT segmentation, despite not adversely affecting tumor measurements. Of the various artifacts known for breast MRI [28,29,30], field inhomogeneity might be alleviated by using the N4ITK algorithm for bias correction. In this study, we investigated the efficacy of the N4ITK algorithm on the automated FGT segmentation quality in breast MRI. In our comparative visual assessment, the bias-corrected BPE mask showed better agreement with the gold standard than the uncorrected BPE mask, with a substantial difference for 6.3–8.0% of the examinations. This means that the FGT segmentation quality grades increased from poor to adequate or from adequate to good by means of bias-correction. Bias corrected BPE masks had significantly higher BPE measurements than uncorrected BPE masks, although voxel counts did not show statistically significant differences. This might suggest that FGT segmentation after bias correction increased the number of voxels correctly identified as fibroglandular tissue while reducing the number of voxels incorrectly identified.

Our recent study by Li et al. [13] demonstrated that addition of BPE to functional tumor volume, longest diameter and sphericity in a multi-feature analysis showed an improvement in pCR prediction over individual features. Another study from our group by Onishi et al. [14] showed the association between lack of BPE suppression and inferior treatment outcome after NAC. These studies illustrate the possible utility of BPE as a biomarker for predicting pathologic outcomes. In these studies, however, a large percentage (about 30–50%) of available MRI examinations were excluded from analyses because of poor segmentation quality even after bias correction. Thus, further refinement of the automated FGT segmentation method is required to take full advantage of BPE in predicting pCR. Additionally, this limitation may be partially due to image quality issues other than field inhomogeneity in the study cohort, in which patients up until 2016 were included in the analyses. Since then, the I-SPY 2 trial has been continually improving image quality. The automated BPE calculation method will continue to be tested in newer cohorts.

This study had several limitations. First, our mean BPE metric is likely to be insensitive to spatial heterogeneity over the entire fibroglandular tissue. In a study by Giess et al., BPE was found to have an asymmetric distribution with higher BPE at peripheral areas due to the arterial blood supply to the breast [31]. By taking the mean overall voxels, we are losing information about spatial BPE patterns. However, since accurate FGT segmentation has a direct impact on the subsequent accuracy of BPE quantification, this study focused on the segmentation aspect. A second limitation is that our dataset is from a multi-center study and some exams may have already been bias corrected using full field-of-view methods. We do not have complete information for the time frame of studies used in this study of whether prior bias correction was performed. This could lessen the effect of our post-process bias correction performed over the restricted bilateral volume. An additional limitation of the FCM method is the a priori selection of the number of clusters. Breast tissue density varies widely in the study population and selection of the first two out of six predefined clusters may not have been optimal for all patients.

To address a few of these limitations, our next steps are to look at other methods for quantifying BPE from FGT segmentations, such as looking at quadrants or other labeled regions of the breast and applying radiomic approaches to better capture heterogeneity. Instead of a single mean BPE metric, additional information about BPE kinetics and spatial patterns may help with outcome prediction for treatment response. As deep learning approaches for image segmentation have become increasingly effective in recent years, we plan to implement such models to obtain better whole breast and tissue segmentations which in turn may give us more accurate BPE estimates.

In conclusion, our study showed that volume-restricted bias field inhomogeneity correction can improve tissue segmentation quality and thus may help further improve quantitative BPE measurements. Exclusion of examinations with poor FGT segmentation leads to an overall smaller sample size and may limit the utility of BPE as a predictor of pCR. Therefore, it is important to improve segmentation accuracy without adverse impact on the yield of examinations for which BPE can be successfully measured. Continued research for improved BPE metrics is in progress and BPE may become a stronger predictor in our future studies.

## Figures and Tables

**Table 1:** Comparative Visual Assessment of Uncorrected vs. Bias-corrected BPE masks.

Assessment	Label for UC	Label for BC	Category
A >> B	A	B	−2: worse agreement for BC than UC with substantial difference *
A > B	A	B	−1: worse agreement for BC than UC
A = B	A	B	0: BC and UC showed equivalent agreement with the gold standard
A < B	A	B	1: better agreement for BC than UC
A << B	A	B	2: better agreement for BC than UC with substantial difference *
A >> B	B	A	2: better agreement for BC than UC with substantial difference *
A > B	B	A	1: better agreement for BC than UC
A = B	B	A	0: BC and UC showed equivalent agreement with the gold standard
A < B	B	A	−1: worse agreement for BC than UC
A << B	B	A	−2: worse agreement for BC than UC with substantial difference *

UC = uncorrected BPE mask, BC = bias-corrected BPE mask. * The difference between UC and BC was equivalent to one or two grades difference as defined in the initial quality assessment.

**Table 2:** Patient Characteristics.

Parameter		Whole Cohort (*n* = 735)	High-Quality Cohort (*n* = 340)	Non-High-Quality Patients (*n* = 395)	*p* Value
Age (y)					
	Mean ± SD	49 ± 11	49 ± 10	49 ± 11	0.898
	Range	24–77	24–77	25–73	
Menopausal status					0.942
	Pre-menopausal	342 (47)	153 (45)	189 (48)	
	Peri-menopausal	26 (4)	12 (4)	14 (4)	
	Post-menopausal	223 (30)	105 (31)	118 (30)	
	Unclear *	95 (13)	47 (14)	48 (12)	
	No data	49 (7)	23 (7)	26 (7)	
Race					0.359
	White	597 (81)	281 (83)	316 (80)	
	African American	78 (11)	28 (8)	50 (13)	
	Asian	47 (6)	23 (7)	24 (6)	
	American Indian or Alaska Native	3 (0)	2 (1)	1 (0)	
	Native Hawaiian or Pacific Islander	4 (1)	2 (1)	2 (1)	
	Mix	6 (1)	4 (1)	2 (1)	
Immunohistochemical subtype					0.667
	HR+/HER2–	299 (41)	140 (41)	159 (40)	
	HR+/HER2+	112 (15)	57 (17)	55 (14)	
	HR–/HER2+	61 (8)	27 (8)	34 (9)	
	HR–/HER2–	263 (36)	116 (34)	147 (37)	
Assigned chemotherapy					0.720
	Standard chemotherapy	158 (21)	71 (21)	87 (22)	
	Experimental chemotherapy	577 (79)	269 (79)	308 (78)	
Treatment response					0.353
	pCR	258 (35)	113 (33)	145 (37)	
	non-pCR	477 (65)	227 (67)	250 (63)	

Unless otherwise specified, data represent the number of patients and data in parentheses are percentages. *p* values show the results of the comparisons between the high-quality cohort vs. the non-high-quality patients. The Mann–Whitney U test was used for continuous variables (i.e., age), and Fisher’s exact test was used for categorical variables. * Unclear because of estrogen replacement therapy or prior gynecological surgery. SD = standard deviation, HR = hormone receptor, HER2 = human epidermal growth factor receptor 2, pCR = pathologic complete response.

**Table 3:** Voxel count before and after bias-correction.

Cohort and Timepoint	Voxel Count for UC BPE Mask	Voxel Count for BC BPE Mask	Difference of Voxel Count *		
			Estimated Pseudo-Median	95% CI	*p*-Value
Whole cohort					
T0	62,791 [39,090, 92,646]	62,372 [37,478, 94,399]	493.5	−357, 1374.5	0.251
T1	58,831 [34,199, 89,447]	58,343 [35,580, 91,010]	693.5	−100.5, 1493	0.086
T2	53,996 [33,076, 85,252]	52,834 [33,168, 83,312]	2.5	−739.5, 770.5	0.995
High-quality cohort					
T0	59,190 [37,981, 87,995]	60,343 [37,233, 87,997]	−310.5	−1293.5, 731	0.565
T1	56,245 [36,219, 83,899]	55,510 [35,830, 83,418]	326.48	−657, 1305.5	0.519
T2	51,346 [34,779, 77,317]	51,124 [33,884, 80,426]	−348	−1247.5, 629	0.455

Data for voxel count show median along with the first and third quartile. BPE = background parenchymal enhancement, UC = uncorrected, BC = bias-corrected. * Voxel count for BC BPE mask minus voxel count for UC BPE mask.

**Table 4:** BPE measurement before and after bias-correction.

Cohort and Timepoint	BPE Measurement for UC BPE Mask	BPE Measurement for BPE Mask	Difference of BPE Measurement *		
			Estimated Pseudo-Median	95% CI	*p* Value
Whole cohort					
T0	23.3 [16.3, 34.3]	24.0 [16.6, 35.1]	0.64	0.52, 0.76	<0.001 **
T1	19.1 [13.5, 27.4]	19.9 [14.1, 28.6]	0.58	0.48, 0.69	<0.001 **
T2	17.1 [12.3, 23.4]	17.6 [12.6, 24.3]	0.48	0.38, 0.58	<0.001 **
High-quality cohort					
T0	23.2 [16.5, 35.1]	23.4 [16.3, 35.1]	0.43	0.29, 0.58	<0.001 **
T1	19.7 [14.5, 27.7]	19.9 [15.2, 27.6]	0.41	0.28, 0.55	<0.001 **
T2	17.7 [13.4, 24.1]	18.1 [13.7, 25.3]	0.36	0.24, 0.49	<0.001 **

Data for BPE measurement show median along with the first and third quartile. BPE = background parenchymal enhancement, UC = uncorrected, BC = bias-corrected. * BPE measurement for BC BPE mask minus BPE measurement for UC BPE mask. ** *p*-value < 0.05.

## Data Availability

The MRI data analyzed in this study is being deposited and will be publicly available in NCI The Cancer Imaging Archive (TCIA): https://www.cancerimagingarchive.net/ (accessed on 18 March 2022) in Spring 2022. In the interim, please contact the corresponding author with data queries.
